# Mechanisms of Invasion Resistance of Aquatic Plant Communities

**DOI:** 10.3389/fpls.2018.00134

**Published:** 2018-02-09

**Authors:** Antonella Petruzzella, Johan Manschot, Casper H. A. van Leeuwen, Bart M. C. Grutters, Elisabeth S. Bakker

**Affiliations:** Department of Aquatic Ecology, Netherlands Institute of Ecology (NIOO-KNAW), Wageningen, Netherlands

**Keywords:** biotic resistance, diversity-resistance hypothesis, niche partitioning, functional group identity, limiting similarity, sampling effect, species diversity, species richness

## Abstract

Invasive plant species are among the major threats to freshwater biodiversity. Few experimental studies have investigated whether native plant diversity can provide biotic resistance to invaders in freshwater ecosystems. At small spatial scales, invasion resistance may increase with plant species richness due to a better use of available resources, leaving less available for a potential invader (Complementarity effect) and/or the greater probability to have a highly competitive (or productive) native species in the community (Selection effect). In submerged aquatic plant communities, we tested the following hypotheses: (1) invader establishment success is greatest in the absence of a native plant community; (2) lower in plant communities with greater native species richness, due to complementary and/or selection effects; and (3) invader establishment success would be lowest in rooted plant communities, based on the limiting similarity theory as the invader is a rooted submerged species. In a greenhouse experiment, we established mesocosms planted with 0 (bare sediment), 1, 2, and 4 submerged plant species native to NW Europe and subjected these to the South African invader *Lagarosiphon major* (Ridl.) Moss. We used two rooted (*Myriophyllum spicatum* L., *Potamogeton perfoliatus* L.) and two non-rooted native species (*Ceratophyllum demersum* L., *Utricularia vulgaris* L.) representing two distinct functional groups considering their nutrient acquisition strategy which follows from their growth form, with, respectively, the sediment and water column as their main nutrient source. We found that the presence of native vegetation overall decreased the establishment success of an alien aquatic plant species. The strength of this observed biotic resistance increased with increasing species richness of the native community. Mainly due to a selection effect, the native biomass of mixed communities overyielded, and this further lowered the establishment success of the invader in our experiment. The strongest biotic resistance was caused by the two native plant species that were of the same functional group, i.e., functionally most similar to the invader. These results support the prediction of Elton’s biotic resistance hypothesis in aquatic ecosystems and indicate that both species richness and functional group identity can play an important role in decreasing establishment success of alien plant species.

## Introduction

Aquatic plants have been crossing geographic barriers mainly due to the ornamental and aquarium trade, and have been intentionally or accidentally introduced to many new aquatic systems (Hussner, 2012). Invasive species are among the major threats to freshwater biodiversity, strongly affecting the structure and functioning of these ecosystems (Dudgeon et al., 2006; Strayer, 2010). As a consequence, there is a growing interest in understanding the factors regulating the success or failure of alien species and what makes plant communities more susceptible or resistant to invasions (Lonsdale, 1999; Davis et al., 2000; Chadwell and Engelhardt, 2008; Thomaz et al., 2015 and references therein).

The biotic resistance hypothesis proposed by Elton (1958) predicts that species-richer communities are more resistant to invasions than species-poorer communities. At small spatial scales, this hypothesis has been largely supported (Kennedy et al., 2002; Lindig-Cisneros and Zedler, 2002; Michelan et al., 2013). However, opposite patterns are observed at larger spatial scales, where species-richer communities are not more resistance to invasion (Stohlgren et al., 1999; Levine, 2000). This ‘paradox’ has been attributed to the relative contribution of extrinsic factors to invasion resistance, such as resource heterogeneity, climate, and disturbance, which can vary considerably with resident native diversity across broad spatial scales (Davis et al., 2000; Naeem et al., 2000). In contrast, at smaller scales, species interactions, such as competition, might play a major role in community assembly and invasion resistance (Fargione et al., 2003).

The diversity of native plant communities can influence the degree of competitive resistance through three different mechanisms. First, multiple species (or functional groups) with non-overlapping resource use strategies can complement each other in the use of available resources, i.e., the complementarity effect. More efficient resource use leaves fewer resources available to potential invader species (Naeem et al., 2000; Britton-Simmons, 2006; Stachowicz et al., 2007; Brown and Rice, 2010; Byun et al., 2013). Second, more diverse communities have a higher probability to have a better competitor or more productive species present, a mechanism known as the sampling or selection effect (Wardle, 2001). These superior competitive species would competitively suppress the invasiveness of alien species. A third alternative mechanism is that more diverse communities also increase the probability of including greater functional diversity and a functionally similar invader (i.e., limiting similarity) (Xu et al., 2004; Hooper and Dukes, 2010). Limiting similarity theory predicts that the species that are most similar to an invader provide the greatest invasion resistance due to niche overlap in resource use. It is important to note that these three mechanisms are not mutually exclusive and can work synergistically (Fargione and Tilman, 2005). Although the patterns and the underlying mechanisms have been widely debated in terrestrial and marine systems, few experimental studies have investigated biotic resistance or its underlying mechanisms in freshwater ecosystems (Kimbro et al., 2013; Michelan et al., 2013; Alofs and Jackson, 2014).

Here, we performed a small-scale and full factorial experiment in which we manipulated native species richness and functional group identity to explore by which mechanisms the diversity of native plant species may decrease the establishment success of aquatic invasive species. Invader establishment success was measured as colonization ability and growth, defined as biomass increase. We used curly-leaved waterweed, *Lagarosiphon major* (Ridl.) Moss as our model species, which is a highly invasive submerged rooted macrophyte in freshwater submerged plant communities (Hussner, 2012). We tested the following three hypotheses: (1) invader establishment success is greatest in the absence of a native plant community (i.e., no resistance present); (2) invader establishment success is lower in plant communities with greater native species richness, due to complementary and/or selection effects; and (3) invader establishment success would be lowest in rooted plant communities, based on the limiting similarity theory as the invader is a rooted submerged species. Interspecific competition should be strongest between functionally similar species.

## Materials and Methods

### Plant Material

The experimental native plant communities were established using four co-occurring submerged aquatic macrophyte species native to, and widely distributed in, Northwestern Europe. We used two rooted [*Myriophyllum spicatum* L. (Haloragaceae), *Potamogeton perfoliatus* L. (Potamogetonaceae)] and two non-rooted species [*Ceratophyllum demersum* L. (Ceratophylaceae), *Utricularia vulgaris* L. (Lentibulariaceae)]. The two distinct groups (rooted and non-rooted) are functionally and morphologically different in their nutrient acquisition strategy which follows from their growth form, with, respectively, the sediment and water column as their main nutrient source. Most rooted species are able to take up nutrients from both the water and the sediment with the relative contribution of each source depending on the relative nutrient availability (Feijoó et al., 2002 and references therein). For submerged rooted plants, the sediment becomes the main nutrient acquisition source once there are available nutrients in it. Non-rooted species absorb nutrients from the surrounding water. Whereas *C. demersum* forms anchoring adventitious roots, its nutrient uptake is almost entirely foliar (Denny, 1972), as well as for *U. vulgaris*, which can absorb nutrients over the entire shoot surface (Friday, 1992).

We selected the South African species *L. major* (Hydrocharitaceae) as invader. *L. major* is a rooted, submerged plant species recognized as a noxious aquatic weed (Hussner, 2012). According to the European and Mediterranean Plant Protection Organization (EPPO), it is present in 13 countries in Europe and listed on the ‘EPPO^1^ List of invasive alien plants’.

*M. spicatum, P. perfoliatus, C. demersum*, and *L. major* were acquired from a commercial plant trader in the Netherlands (De Zuurstofplantgigant, 51°22′00.7″N, 5°15′0.7.2″E). *U. vulgaris* was collected from a private pond in Netherlands (52°09′5″N, 5°00′16″E). All plants were pre-cultivated under controlled greenhouse conditions with a 16/8 h light/dark cycle at an average temperature of 21°C during the day and 16°C during the night. We used 200 L cattle tanks (two tanks per species) filled with 20 L of artificial plant pond sediment (Plant soil Moerings – Velda), 20 L of washed sand on top and filled with tap water (mean ± SD, *n* = 6: 20 ± 3.2 μg L^-1^ P-PO_4_; 37.7 ± 3.1 μg L^-1^ N-NO_3_; 1 ± 1.7 μg L^-1^ N-NO_2_; 13 ± 31.8 μg L^-1^ N-NH_4_, pH 8.20 ± 0.02). Plants were cultivated for at least 2 weeks.

### Experimental Design

To evaluate the effects of native community diversity on the invader establishment success of *L. major*, we established 72 mesocosms in a greenhouse under controlled conditions (with a set regime of 16/8 h light/dark, 16–21°C night/day) at Netherlands Institute of Ecology (NIOO-KNAW) during the summer of 2016 (July–September) in Netherlands. The experiment ran for 10 weeks including 2 weeks of establishment of the native plant community.

For the experiment, 147 non-rooted apical shoots without lateral shoots were collected from the cultivation tanks for each of the species *M. spicatum, P. perfoliatus* and *C. demersum*; for *U. vulgaris* the entire plant was used instead of only the apical part. The shoots were cut to be 15 cm long and washed in running tap water to remove any material attached. Of the 147 shoot fragments of each species, 15 were randomly selected, dried to a constant mass at 60°C for at least 48 h, and weighed for initial biomass measurements. The plant density in the mesocosms was kept constant across treatments at 8 shoots per mesocosm, which is 254.78 plants m^-2^. Thus, mesocosms containing two species contained four individuals of each species, whereas those with four species contained two individuals of each species. Such shoot densities are within the range of the shoot densities of submerged macrophyte communities in natural conditions (Li et al., 2015). The plant shoots were planted 5 cm deep in the sediment.

The mesocosms consisted of 10 L white plastic buckets (32.5 cm height and 20 cm diameter) filled with a bottom layer of artificial plant pond sediment (Plant soil Moerings – Velda) (1 cm/ 200 g, organic matter content = 34.31%) with a top layer of washed sand (0.8–1.0 mm grain size; 5 cm deep, 2650 g sand per bucket, organic matter content = 0.16%)., Then the bucket was filled up with tap water (mean ± SD, *n* = 6: 20 ± 3.2 μg L^-1^ P-PO_4_; 37.7 ± 3.1 μg L^-1^ N-NO_3_; 1 ± 1.7 μg L^-1^ N-NO_2_; 13 ± 31.8 μg L^-1^ N-NH_4_). The water level was maintained constant during the whole experiment by refilling once a week to compensate for evapotranspiration. The physical and chemical parameters of the water were measured weekly in all mesocosms and the growing conditions were found to be suitable for the fragments (mean values at daytime throughout the experiment, mean ± SD, *n* = 792: water temperature 22 ± 0.9°C, dissolved oxygen concentration 7.9 ± 2.0 mg L^-1^, pH 9.6 ± 0.6, *n* = 432: alkalinity 0.8 ± 0.5 mEq L^-1^).

Treatments were applied in a full factorial design (see **Table 1**). We established four levels of native plant species richness, 0 (bare sediment), 1 (monocultures), 2, and 4 species (see **Table 1**). To test limiting similarity, we grouped our treatments according to plant growth form, assuming this parameter is also related to the major nutrient acquisition sources (the sediment and the water column) and therefore intensity of nutrient competition. Our plant species represented two different functional groups: a rooted group containing only rooted submerged macrophytes and a non-rooted group containing only non-rooted ones. When both functional groups were combined in one bucket, we defined these as mixtures. Each of the 12 treatments (4 species richness levels, 3 functional group combinations) was replicated 6 times, using a block design, yielding a total of 72 mesocosms (**Table 1** and Supplementary Figure 1).

**Table 1:** Overview of the 12 treatments used in the mesocosm experiment.

Diversity treatment	Community composition	Species	Functional group(s)	Species richness
1	Bare sediment	Bare sediment		0
2	M	*Myriophyllum spicatum*	Rooted	1
3	P	*Potamogeton perfoliatus*	Rooted	1
4	C	*Ceratophyllum demersum*	Non-rooted	1
5	U	*Utricularia vulgaris*	Non-rooted	1
6	M _+_ P		Rooted	2
7	C _+_ U		Non-rooted	2
8	M _+_ C		Mixture	2
9	M _+_ U		Mixture	2
10	P _+_ C		Mixture	2
11	P _+_ U		Mixture	2
12	M _+_ P _+_ C _+_ U		Mixture	4

*Functional groups are classified based on the main nutrient acquisition strategy which follows from their growth form.*

After the establishment of the native plant community (determined by the growth of new shoots of at least one individual of each species, which took 2 weeks), we introduced two *L. major* propagules per mesocosm. Each propagule consisted of a 15-cm-long shoot with apical tip, but no lateral branches, to minimize variation in the size of the initial shoot material. A total of 15 *L. major* fragments were randomly selected, dried to a constant mass at 60°C for at least 48h, and weighed for initial biomass measurements. Fragments with an apical tip have higher regeneration and colonization abilities and higher growth rates than fragments without apical tips (Riis et al., 2009). Invader fragments were not planted in the sediment but were dropped into the mesocosms simulating how alien species arrive in a new area where a native community is already established (Riis and Sand-Jensen, 2006). Pilot trials showed that the fragments float for days to weeks first growing side branches. Gradually they start forming aerial roots and growing downwards to sink, thus, reaching the sediment (Supplementary Figure 2).

### Plant Harvest and Data Collection

We measured invader establishment success in terms of the ability of the fragments to colonize and grow. We defined successful invader colonization as at least one fragment of the invader having its roots attached in the sediment (Supplementary Figure 2). At the end of the experiment, we counted the number of mesocosms that was successfully colonized. Invader growth was defined as biomass increase (dry weight, DW). At the end of the experiment (after 8 weeks), the invader was harvested. Total root and shoot DW were determined summing values from both introduced fragments. From these values, we calculated the root:shoot ratio and relative growth rate (RGR). The RGR was calculated considering the total biomass of the invader (including both fragments and roots + shoots) in the mesocosms as follows:

$$\begin{array}{c} RGR=(\ln W_{f}-\ln W_{i})/{\mathrm{day}}^{-1}; \end{array}$$

where, *W*_f_ = final DW; *W*_i_ = initial DW.

Additionally, the native plant community biomass was harvested and sorted by species at the end of the experiment. All the harvested plants were dried to a constant mass at 60°C for at least 48 h, and weighed.

### Data Analyses

We analyzed the ability of invader fragments to colonize, defined as the invader having at least one fragment with roots attached in the sediment, using Fisher’s exact test, which is used to assess the significance of a difference between the proportions in two groups.

To test the effects of the native community on the invading species, we used a mixed-modeling approach. Response variables of interest were the root DW, shoot DW, root:shoot ratio, and RGR of the invading species *L. major*. We used General Linear Mixed-effects Models (General LMMs) in case the response variables were normally distributed, i.e., all data for *L. major* shoot DW and RGR, and the data for *L. major* root DW and root:shoot ratio when analyzed in combination with only native species monocultures. We used Generalized Linear Mixed-effects Models (Generalized LMMs) in case of non-normal distributions, i.e., when including all species richness treatments for *L. major* root DW and root:shoot ratio. Fixed factors that were included in the models were (1) the presence of native communities (bare sediment and the 4 species monocultures), (2) species richness (0, 1, 2, or 4 species), or (3) functional group identity (bare sediment, non-rooted, rooted, or a mixture of non-rooted and rooted native species present). Block (the 6 replicates) was included as random factor in all models. Normality of residuals and homoscedasticity were checked with plots of residual versus fitted values, and qqplots of residuals. Response variables were natural log-transformed when necessary for satisfying assumptions of normality. We assessed statistical significance of fixed factors in the models by performing likelihood ratio tests between models including and excluding these factors (*F*-tests for General LMMs and Chi-squared tests for Generalized LMMs). All statistical analyses were carried out using RStudio version 3.4.2 (R Core Team, 2017).

To test our first hypothesis that *L. major* establishment success would be greatest in absence of a native community, we used a General LMM [*lme* function in the R package *nlme* (Pinheiro et al., 2017)] to test whether all four invader response variables differed between the treatment with only bare sediment (as intercept) and each of the four monocultures. Root DW and root:shoot ratio were natural log-transformed for satisfying assumptions of normality.

To test our second hypothesis that invader establishment success would be lowest in richer native plant communities, we tested the effect of species richness on all four invader response variables. Possible effects of species richness on *L. major* root DW and root:shoot ratio were analyzed following the procedure described by Fletcher et al. (2005). This method is applied for ecological data that has a substantial proportion of zeros and is positively skewed which often makes assumptions for linear analysis (e.g., normality of errors) invalid. First, we analyzed whether invader root formation (presence/absence) was affected by species richness by fitting a binomial Generalized LMM [*glmer* function in the R package *lme4* (Bates et al., 2015)] using presence or absence of roots as response variable. Second, for those mesocosms in which the invader developed roots, we subsequently analyzed possible effects of species richness on the amount of root biomass produced using the *lmer* function of the same package. We natural log-transformed root DW and root:shoot ratio to ensure normality of model residuals. Possible effects of species richness on normally distributed *L. major* shoot DW and RGR were assessed using General LMMs. To test the effects of species richness on the native community biomass, we also used a General LMM, in which species richness was a fixed factor, native community biomass the response variable and block as random factor.

To test our third hypothesis that invader establishment success would be lowest in rooted submerged species based on limiting similarity theory, we analyzed whether the four invader response variables were affected by functional group identity. We fitted four mixed models with functional group identity as fixed factor and block as random factor. For the root and root:shoot ratio data, we also analyzed following the procedure described by Fletcher et al. (2005) but we are only interested in the results of those mesocosms in which the invader developed roots. Root DW and root:shoot ratio given the presence of roots were natural log-transformed for satisfying assumptions of normality. For models with normally distributed shoot DW and RGR as response variables, we used General LMMs. We used Tukey’s *post hoc* test to detect pairwise differences between functional group identity treatments. We applied a Bonferroni’s correction for adjusting the significance levels to control for Type I error in a multiple testing situation.

### Additive Partitioning of Diversity Effects

To determine the mechanisms of possible biodiversity effects, the net diversity effect (Δ*Y*) in 4 species mixture treatment was partitioned into a complementarity effect and selection effect using the additive partitioning of biodiversity effects method proposed by Loreau and Hector (2001). This method compares the total mixture yield (*Y_O_*) with the expected yield of the mixtures based on monoculture yields of component species (*Y_E_*).

$$\begin{array}{c} \Delta Y=Y_{O}-Y_{E} \end{array}$$

The complementarity effect for a specific number of species (*N*) was calculated as:

$$\begin{array}{c} N\bar{\Delta RY}\bar{M} \end{array}$$

where $\bar{\Delta RY}$ is the average change in expected relative yield of all species in the mixture and $\bar{M}$ is the average monoculture yield. A positive complementarity effect occurs if species yields in a mixture are on average higher than expected based on monoculture yields of component species (Loreau and Hector, 2001).

The selection effect was calculated as the covariance between monoculture yields of component species (*M*) and their change from expected relative yield in the mixture of all species in the mixture (ΔRY) multiplied by *N* of the mixture.

$$\begin{array}{c} N\;\mathrm{cov}(\Delta RY,M) \end{array}$$

A positive selection occurs if species with higher than average monoculture yields dominate the mixtures (Loreau and Hector, 2001).

## Results

Overall, the presence of native plants strongly decreased the ability of the invader to colonize. The invading species *L. major* had fragments with roots attached to the sediment in only 5 out of 66 mesocosms in which native plants were present (7.6 % colonized). Whereas in the treatment with bare sediment invader fragments attached with roots to the sediment in 4 out of 6 mesocosms (66.7% colonized, which was significantly higher than when native plants were present, odds ratio = 0.045, *p* = 0.002). When native plants were present, in particular treatments with *U. vulgaris* species were colonized by *L. major* fragments. In *U. vulgaris* monocultures, 50% of the mesocosms were colonized and this rate decreased when *U. vulgaris* grew in combination with other species to 16.7% when grown with either *P. perfoliatus* (PU) or *C. demersum* (CU), whereas no colonization was observed when *U. vulgaris* was grown with *M. spicatum* (MU).

Generally, *L. major* formed significantly more shoot biomass in the absence of a native plant community (**Table 2**). Contrasts between the bare sediment treatment and monocultures of the native plants showed that *L. major* root biomass was significantly larger in the bare sediment treatment than in monocultures of the two rooted species *M. spicatum* and *P. perfoliatus*, but not in non-rooted *C. demersum* and *U. vulgaris* (**Table 2**). Similarly, we also did not find significant differences between root:shoot ratio and RGR of the invader in the bare sediment treatment and monocultures of *C. demersum* and *U. vulgaris* (**Table 2**), i.e., invader growth was as great in the non-rooted species treatments as in the absence of native plants. In contrast, monocultures of the rooted species *M. spicatum* and *P. perfoliatus* significantly lowered the invader’s root:shoot ratio and RGR (**Table 2**). It is important to point out, at the start of the experiment, all the native plants were growing well, whereas during the course of the experiment *U. vulgaris* plants started dying. These results likely indicate the invader may have benefited from this to colonize and grow.

**Table 2:** Results of general linear mixed effects model (General LMM) using contrasts between the bare sediment mesocosms (set as intercept) and the effects of native species monocultures of *Myriophyllum spicatum* (M), *Potamogeton perfoliatus* (P), *Ceratophyllum demersum* (C), and *Utricularia vulgaris* (U) on root dry weight, shoot dry weight, root:shoot ratio, and relative growth rate (RGR) of the invader *Lagarosiphon major*.

Invader response variable	Treatment	Mean ±*SD, n* = 6	*t*	*p*( >*|t|* )
Root DW (g)^∗^	Bare sediment	**59.42 ± 77.3**		
	M	**0.05 ± 0.1**	**–3.64**	**0.001**
	P	**3.37 ± 2.5**	**–3.04**	**0.006**
	C	14.37 ± 10.2	–1.72	0.100
	U	87.57 ± 118.4	–0.03	0.968
Shoot DW (g)	Bare sediment	**1.71 ± 0.25**		
	M	**0.91 ± 0.21**	**–6.65**	**<0.001**
	P	**1.00 ± 0.16**	**–5.93**	**<0.001**
	C	**1.42 ± 0.18**	**–2.38**	**0.026**
	U	**1.44 ± 0.28**	**–2.25**	**0.035**
Root:shoot^∗^	Bare sediment	**0.033 ± 0.039**		
	M	**0 ± 0**	**–3.08**	**0.005**
	P	**0.003 ± 0.002**	**–2.38**	**0.026**
	C	0.011 ± 0.01	–1.35	0.189
	U	0.058 ± 0.071	0.33	0.741
RGR (g g^-1^ DW day^-1^)	Bare sediment	**0.026 ± 0.003**		
	M	**0.014 ± 0.004**	**–6.81**	**<0.001**
	P	**0.016 ± 0.003**	**–5.77**	**<0.001**
	C	0.022 ± 0.002	–2.08	0.050
	U	0.023 ± 0.004	–1.60	0.123

*Bold numbers indicate significant differences (*p* < 0.05). ^∗^Natural log-transformed variables for satisfying assumptions of normality.*

**FIGURE 1:**
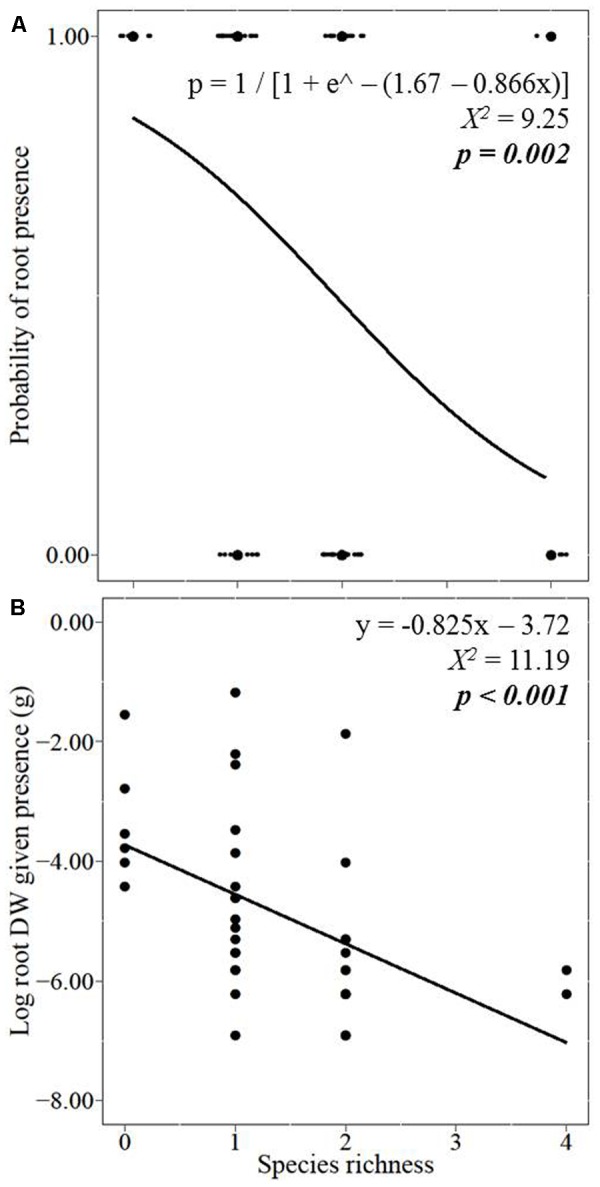
Effect of native plant species richness on the invader *Lagarosiphon major* **(A)** root formation and, given their presence, on **(B)** log-transformed root biomass production. Data points were jittered in the graph **(A)** so the binomial (presence/absence) of roots at many of the treatments could be seen. Significance level at *p* < 0.05.

**FIGURE 2:**
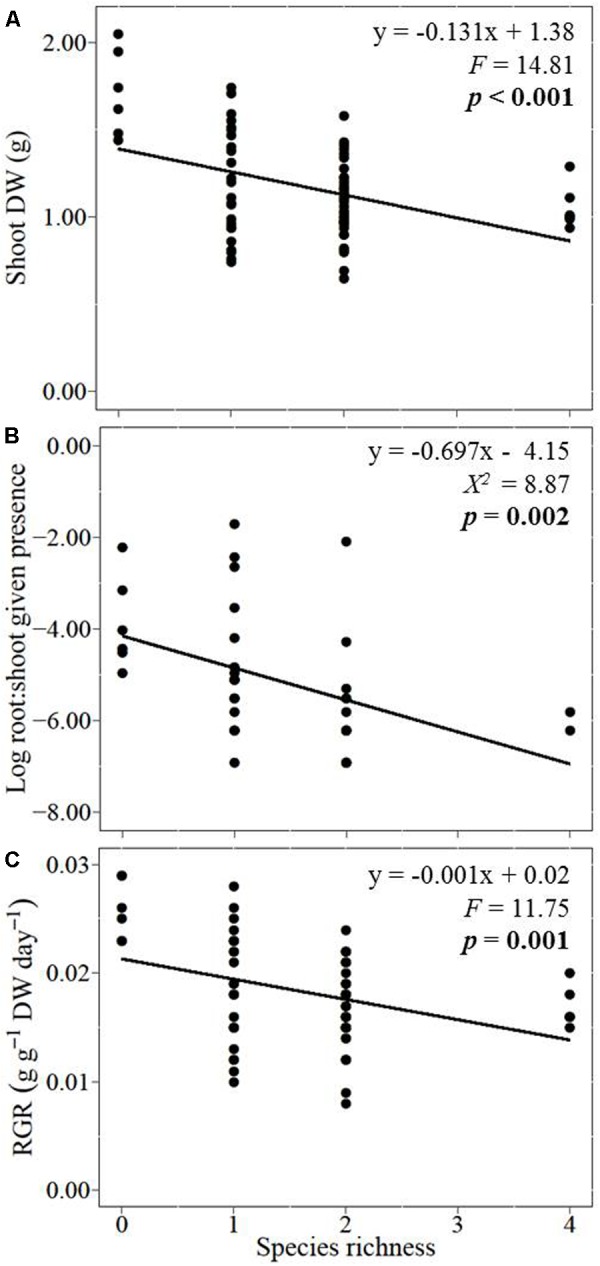
Effects of native plant species richness on the invader *Lagarosiphon major* **(A)** shoot biomass, **(B)** log-transformed root:shoot ratio given their root formation, and **(C)** relative growth rate (RGR). Significance level at *p* < 0.05.

**FIGURE 3:**
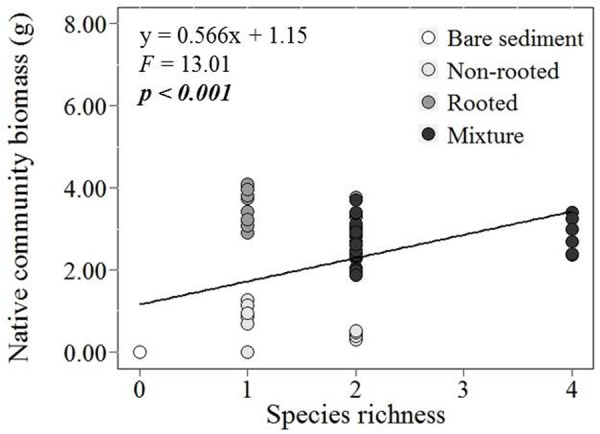
Effect of native plant species richness on total native community biomass production (g DW) per mesocosm.

**FIGURE 4:**
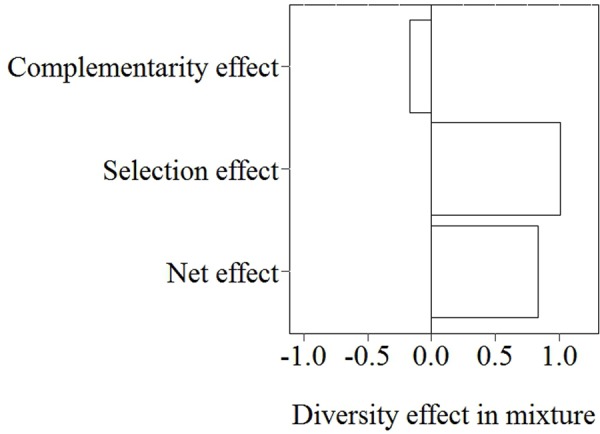
Partitioning of the diversity effect on native community biomass of the highest species richness level (4 species) into a complementarity effect and a selection effect by applying the additive partitioning diversity effect method proposed by Loreau and Hector (2001).

With increasing species richness of the native plant community, the growth of *L. major* decreased. Floating *L. major* fragments were less likely to form roots when native species richness was higher, and when roots were formed, their biomass was lower (**Figures 1A,B**). With increasing native species richness shoot biomass production, root:shoot ratio (given the presence of roots) and RGR of the invader also decreased (**Figures 2A–C**). Native species richness enhanced the biomass of the native community (**Figure 3**). Native biomass at the highest species richness level (4 species) was 29.2% more than the average biomass of monocultures, indicating overyielding. The positive net diversity effect was mainly caused by a selection effect (**Figure 4**), while the complementarity effect was slightly negative. This positive selection effect was most likely caused by the biomass production of the two rooted species, *M. spicatum* and *P. perfoliatus.* These species produced more biomass (mean ± SD, *n* = 12: 3.54 ± 0.43 g) than the two non-rooted *C. demersum* and *U. vulgaris* (mean ± SD, *n* = 12: 0.48 ± 0.52 g, **Figure 3**) in monocultures. Their presence in higher proportion in the mixtures was the main reason for the larger native plant productivity in richer communities.

**FIGURE 5:**
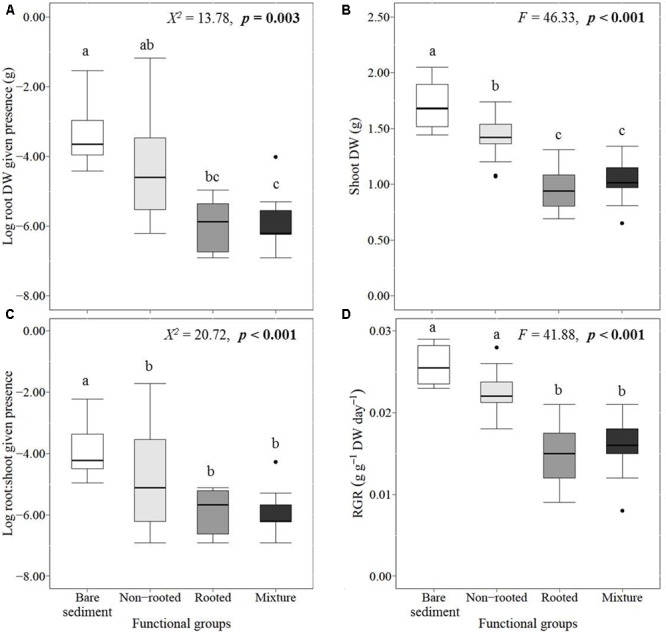
Median, SE (boxes), and minimum and maximum (whiskers) values of (given the presence of roots), **(A)** log-transformed root biomass, **(B)** shoot biomass, **(C)** log-transformed root:shoot ratio, and **(D)** relative growth rate (RGR) of the invader *Lagarosiphon major*. Different lowercase letters indicate statistically significant differences between functional group treatments (Tukey’s *post hoc* test). Significance levels were determined after Bonferroni’s correction (*p* < 0.008).

*L. major* establishment success did depend on which functional groups were present (**Figures 5A–D**). Compared to the bare sediment treatment, *L. major* shoot biomass was significantly decreased in the presence of non-rooted native plants, and even more so in the presence of rooted native plants and mixed communities (**Figure 5B**). The root biomass (given the presence of roots) and the RGR of the invader were most negatively influenced by rooted plants and mixtures, whereas the non-rooted plants did not significantly inhibit the invader’s root biomass and RGR compared to the bare sediment treatment (**Figures 5A,D**). The root:shoot ratio of the invader was significantly lower in all treatments containing native plants, which did not depend on the functional groups present (**Figure 5C**). This may be due to the large variance of invader root biomass, and subsequently root:shoot ratio, observed in the non-rooted plant community treatment. This large variation probably results from contrasting responses of the invader to both non-rooted species, with more root production of the invader in *U. vulgaris* than in *C. demersum* treatments (**Table 1**).

## Discussion

We found that the presence of native vegetation overall can decrease the establishment success of an alien aquatic plant species. The strength of this observed biotic resistance – as proposed by Elton (1958) – increased with increasing species richness of the native community. We could demonstrate that this was mainly due a selection effect, as a result of which the native biomass of mixed communities overyielded, which further lowered the establishment success of the invader in our experiment. The strongest biotic resistance was caused by the two native plant species that were of the same functional group, i.e., functionally most similar to the invader. These results support the prediction of Elton’s (1958) biotic resistance hypothesis and demonstrate that it can be applied to aquatic ecosystems. Moreover, our results show that both species richness and functional group identity can play an important role in decreasing establishment success of alien species.

We expected that invader establishment success, defined as the ability of invader fragments to colonize and grow, would be greatest in the absence of a native plant community. In line with our first hypothesis, the highest colonization rate of *L. major* fragments in bare sediment indicates that the ability of the invader to colonize was highest in the absence of biotic resistance. However, treatments with the species *U. vulgaris* were also colonized by *L. major* fragments to a certain extent, especially in the monoculture. As a result, *L. major* growth was as much in non-rooted species treatments as in bare sediment, with the only exception of shoot biomass. This is likely due to the limited growth of the two non-rooted native species in our experiment. When we started the experiment all the native plants were growing well, whereas during the course of the experiment the rooted plants performed better than the non-rooted ones. In particular, *U. vulgaris* plants started dying, which likely benefited the chance of the invader to colonize and grow. The death of *U. vulgaris* shoots increased the chances of *L. major* fragments reaching the sediment surface due to removal of the physical barrier that these submerged plant biomass imposed (Supplementary Figure 3).

The decrease in *L. major* growth observed with an increase of native species richness supports our second hypothesis. Our results are consistent with previous diversity-invasibility experiments performed in terrestrial and marine ecosystems which also showed a reduced invasibility in more species rich plant communities (Naeem et al., 2000; Fargione and Tilman, 2005; Britton-Simmons, 2006; Byun et al., 2013; Michelan et al., 2013; Zhu et al., 2015). We hypothesized that this species richness effect could be explained by a selection and/or complementarity effect (Naeem et al., 2000; Brown and Rice, 2010; Byun et al., 2013). The partitioning of the biodiversity effect into these two mechanisms showed that the decrease of invader growth would not be explained by native species richness *per se*, but perhaps more specifically by competition between the invader and a particular native species and/or functional group which was indicated by the selection effect. In our study both rooted species, *M. spicatum* and *P. perfoliatus*, which produced more biomass in the monocultures, dominated the mixtures, leading to a high positive selection effect and high biomass and as a result, a lower growth of the invader. Other studies have also shown the effects of the presence of highly competitive species on reducing invader success in terrestrial and marine ecosystems (Wardle, 2001 and references therein; Arenas et al., 2006). However, few studies have experimentally attempted to disentangle the effects of diversity in freshwater ecosystems (Byun et al., 2013; Michelan et al., 2013). In a greenhouse experiment, Michelan et al. (2013) found that the invasiveness of the wetland grass species *Urochloa arrecta* (Hack. ex T. Durand & Schinz) Morrone & Zuloaga was negatively affected by the species richness of native wetland plants, but discarded a potential selection effect because *U. arrecta* growth did not differ in different native species monocultures.

Based on the mechanism of limiting similarity, we expected that invader establishment success would be lowest in rooted plant communities, as the invader is a rooted submerged species. Several studies performed in terrestrial systems have found evidence of functional similarity reducing invader success (Fargione et al., 2003; Pokorny et al., 2005; Mwangi et al., 2007; Young et al., 2009; Hooper and Dukes, 2010), or partly reducing invader success (Price and Paertel, 2013). The results of *L. major* growth partly supported our third hypothesis. Invader shoot biomass and RGR, indeed, were the lowest both in rooted plant communities and in mixtures that were dominated by rooted plants. However, root biomass production of *L. major* was not consistently lower in rooted than in non-rooted native plant communities. This can be explained by the large variation in root production of *L. major* between the treatments with the two non-rooted native plant species. Although *C. demersum* performed less compared to the rooted species, the invader did not produce as much of root biomass as in the *U. vulgaris* treatments, causing large variation in the amount of root production by the invader in the presence of non-rooted species as a functional group.

Several reasons may underlie the negative effects on invader success by native plants. In general, suppression of *L. major* was particularly strong in native communities containing rooted submerged plants. These species produced the most biomass, which is in line with the rapid depletion of pore water nutrients over the sampling weeks (Supplementary Figure 4). Once the nutrients in the sediment are consumed, rooted submerged plants have the ability to take up nutrients also from the water column (Madsen and Cedergreen, 2002 and references therein). This may have caused depletion of nutrients in the water column first in the non-rooted plant treatments and then eventually also in the rooted plant treatments, leaving less available for the invader, thus, limiting its growth (Supplementary Figure 5). This has been demonstrated for the native rooted submerged aquatic plant species *Vallisneria americana* Michaux, which reduced the colonization success of the exotic invasive species, *Hydrilla verticillata* (L.F.) Royle through nutrient draw-dawn in the water column (Chadwell and Engelhardt, 2008). Furthermore, due to their high productivity, densely growing native plants can also prevent colonization and decrease the growth of the invader by acting as a physical barrier, preventing the propagules to reach the sediment surface. Invader propagules might not allocate biomass for colonization, root production, for example, once they are physically trapped in these dense canopies. Alternatively, when the native plants do not form a lot of biomass, such as *U. vulgaris* in our study, invasive fragments can reach the sediment and can start root development, using the excess of nutrients in the sediment (pers. obs. AP). This can thus allow the invader to have access an alternative nutrient source, strongly enhancing its subsequent performance. Additionally, we cannot rule out the possibility that allelopathic effects have occurred, influencing the performance of the invader. In particular, *M. spicatum* and *C. demersum* are known to contain and excrete allelochemicals, which can inhibit the growth of other primary producers (Gross and Bakker, 2012; Grutters et al., 2017). This may have limited the general shoot growth of the invader, or more specific processes, such as the formation of roots. However, allelopathic effects are very difficult to establish (Hilt and Gross, 2008), and hence, the influence of allelopathy in our experiment remains speculative, but cannot be ruled out either.

Our experiment was designed to have the sediment as the main nutrient source, whereas nutrients were not directly added to the water column, but could leak from the sediment into the water column. As such, our experiment represents a situation in which the water layer is transparent and relatively low in nutrients, whereas the sediment is more nutrient rich. This is a rather common situation in many restored shallow water bodies, which often contain nutrient-rich sediment, as a result of eutrophication in the past, whereas the water column is relatively low in nutrients, as a result of measures to reduce nutrient loading, in particular to remove excess phosphorus (e.g., Immers et al., 2015). In these systems, nutrients may be provided to the water column by leaking from the sediment, resulting in internal loading. These systems are sensitive to alien plant invasions, when there are no native plants present. However, native rooted submerged plants can grow very well under these conditions (Verhofstad et al., 2017), which may prevent invasion. If we would have provided nutrients in the water column in our experiment, and not in the sediment, the free submerged plants would have performed much better, and may have outcompeted the invader, whereas the rooted plants may have performed less well, even though nutrients would have precipitated on the sediment. Where the nutrients are more abundant will thus likely affect which functional plant group will dominate, but may not necessarily affect the result that invader establishment is reduced in more species rich native plant communities. Furthermore, it is important to highlight that aquatic plant communities are very often dominated by one or a few vascular species (Engelhardt and Ritchie, 2001), suggesting species or functional group identity is likely to play an important role in biotic resistance to plant invasion in these systems, as we also found in our study.

At large spatial scales, the outcome of invasion success is determined not only by species interactions, but also by the interaction between covarying factors such as propagule pressure and environmental conditions (including disturbance, climate, and resource heterogeneity) (Naeem et al., 2000; Fleming and Dibble, 2015). The conflicting results of plant diversity effects on invasion resistance found at different spatial scales, i.e., ‘paradox,’ have been attributed to the lack of control of these environmental factors (Naeem et al., 2000). Similarly, whereas Elton’s (1958) hypothesis has been largely supported in experiments using pre-defined plant assemblages, conflicting results have been found in field experiments conducted at small spatial scales, which also likely result from the influence of covarying, uncontrolled environmental conditions (Capers et al., 2007). Thus, it is difficult to determine whether the diversity effect on invasion resistance results purely from the species interactions or include the interaction with covarying environmental factors. Controlled mesocosms experiments, such as we performed, allow us to disentangle the role of the effect of species richness, excluding such covariation.

Despite experiments manipulating plant diversity and testing invasion resistance being performed in marine and terrestrial systems (Levine et al., 2004; Kimbro et al., 2013), there are only very few performed in freshwater systems (Alofs and Jackson, 2014). The most recent meta-analysis found only four studies of competitive resistance to an invasive freshwater producer and none of them explicitly addressed diversity (Wakeman and Les, 1994; Dickinson and Miller, 1998; Irfanullah and Moss, 2004; Chadwell and Engelhardt, 2008). We found that native plant diversity increases the biotic resistance of native communities to aquatic plant invaders. This is an important result. Alien species are currently spreading across the globe at unprecedented rates and this process is combined with rapid loss of our native biodiversity (Milligan et al., 2014), which we here show helps to prevent these alien species from becoming invasive. Our study therefore illustrates the importance of studying both invasions and loss of native biodiversity, and especially the novel interactions among native and invasive species. Furthermore, it stresses the importance of the conservation of aquatic plants as a mean to increase the robustness of our aquatic ecosystems, mainly the restored ones.

## Author Contributions

AP, BG, and EB conceived and designed the experiment. AP and JM collected all data. AP, CvL, and BG analyzed the data. AP, JM, CvL, and EB interpreted the data. AP wrote the paper with assistance from CvL and EB. AP, JM, CvL, BG, and EB performed a critical revision and approved this final version of the manuscript to be published.

## Conflict of Interest Statement

The authors declare that the research was conducted in the absence of any commercial or financial relationships that could be construed as a potential conflict of interest.

## Footnotes

1 http://www.eppo.org
