# Supplementary material for: Mechanisms of Invasion Resistance of Aquatic Plant Communities
Source: Front Plant Sci. 2018 Feb 9;9:134. doi: 10.3389/fpls.2018.00134 (PMC5811644; doi:10.3389/fpls.2018.00134)
Supplement: Supplementary file 1 [file Data_Sheet_1.PDF]

Supplementary Material

Mechanisms of Invasion Resistance of Aquatic Plant Communities

Antonella Petruzzella*, Johan Manschot, Casper H. A. van Leeuwen, Bart M. C. Grutters, Elisabeth S. Bakker

*** Correspondence:** Corresponding Author: a.petruzzella@nioo.knaw.nl

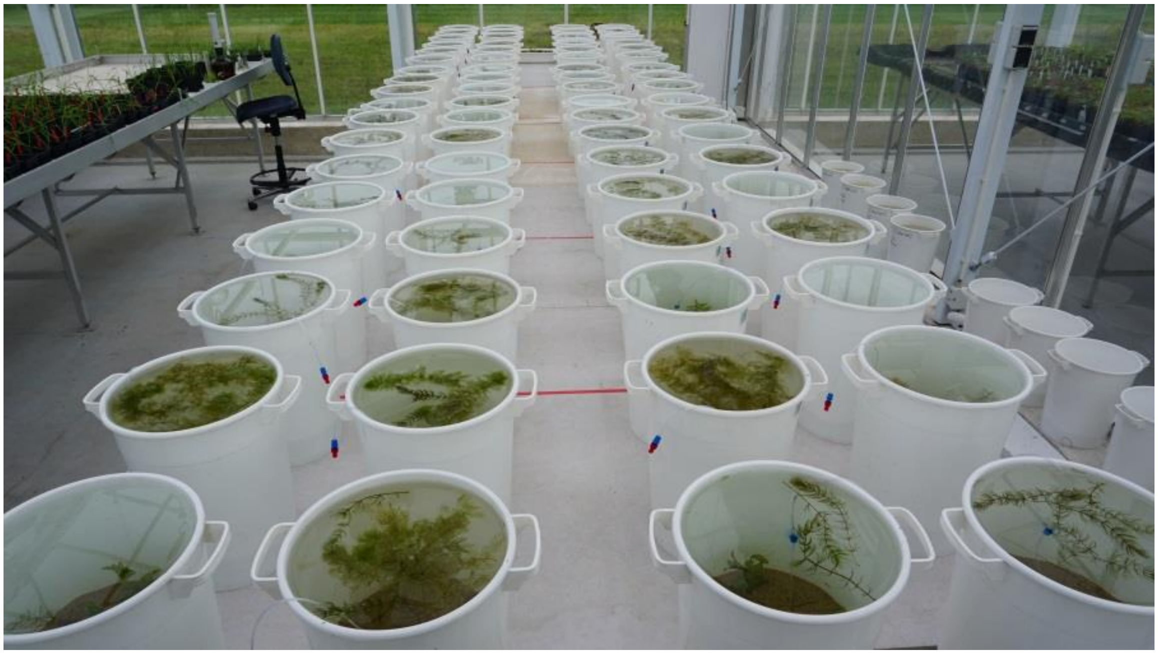

Supplementary Figure 1. Overview of the experiment.

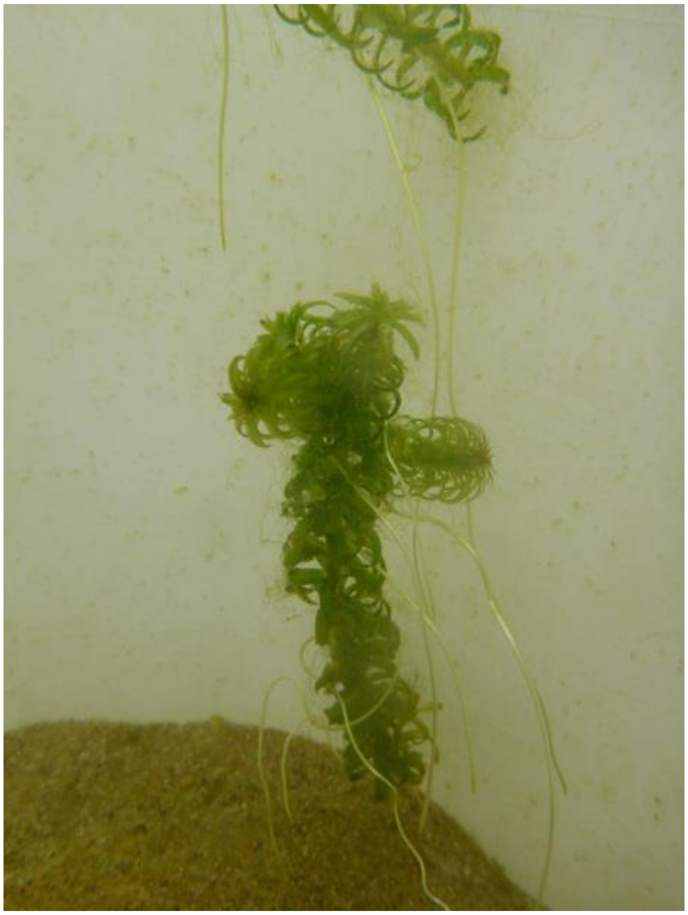

Supplementary Figure 2. Dynamic of *Lagarosiphon major* (Ridl.) Moss propagules settlement in bare sediment. These fragments float for days to weeks, first growing side branches. Gradually they start forming aerial roots and growing downwards to sink, thus, reaching the sediment.

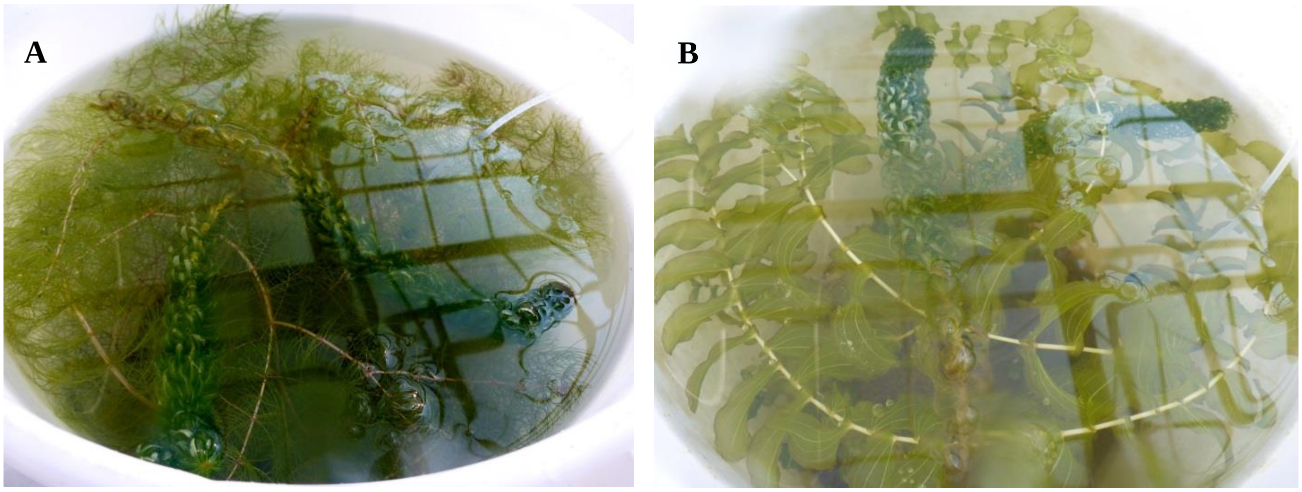

Supplementary Figure 3. Photograph of the monocultures of rooted submerged aquatic macrophytes and *L. major* fragments on top showing the physical barrier imposed by their canopies. **(A)** *Myriophyllum spicatum* L. and **(B)** *Potamogeton perfoliatus* L.

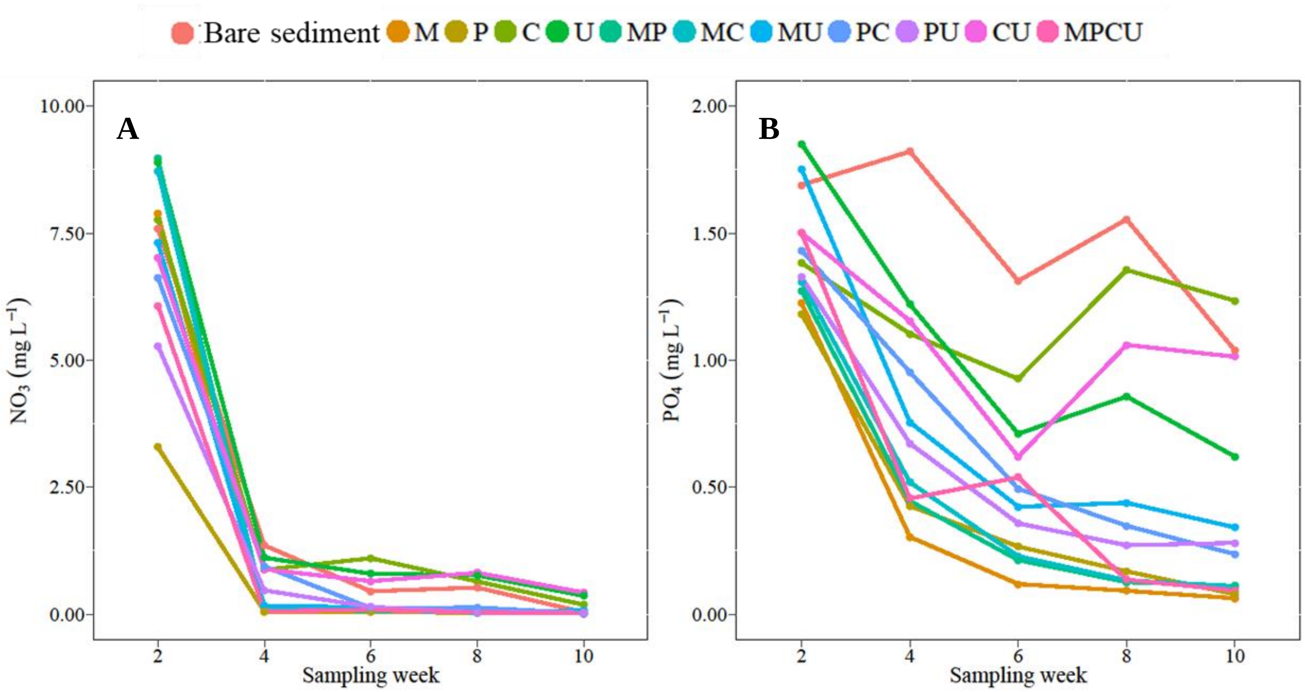

Supplementary Figure 4. Pore water **(A)** nitrate and **(B)** phosphate concentrations of the treatments over the experiment. Values are means per treatment (n=6). Treatments are abbreviated with: Bare sediment, M- *Myriophyllum spicatum* L., P- *Potamogeton perfoliatus* L., C- *Ceratophyllum demersum* L. and U- *Utricularia vulgaris* L. in a full factorial design. Samples were collected every two weeks after the start of the experiment.

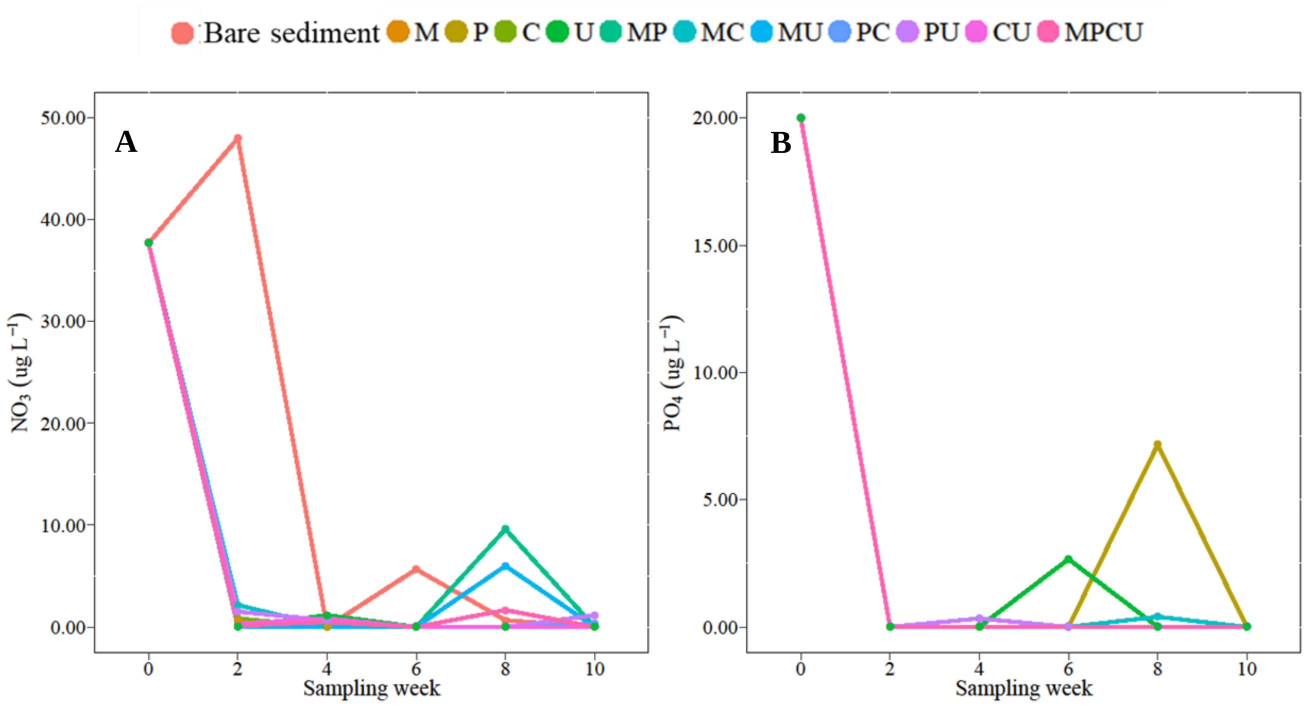

Supplementary Figure 5. Water column **(A)** nitrate and **(B)** phosphate concentrations in the treatments over the experiment. Values are means per treatment (n=6). Treatments are abbreviated with: Bare sediment, M- *Myriophyllum spicatum* L., P- *Potamogeton perfoliatus* L., C- *Ceratophyllum demersum* L. and U- *Utricularia vulgaris* L. in a full factorial design. Samples were collected at the start and every two weeks of the experiment.
